# Epidemiological and economic impact of pandemic influenza in Chicago: Priorities for vaccine interventions

**DOI:** 10.1371/journal.pcbi.1005521

**Published:** 2017-06-01

**Authors:** Nargesalsadat Dorratoltaj, Achla Marathe, Bryan L. Lewis, Samarth Swarup, Stephen G. Eubank, Kaja M. Abbas

**Affiliations:** 1 Department of Population Health Sciences, Virginia Tech, Blacksburg, VA, United States of America; 2 Network Dynamics and Simulation Science Lab, Biocomplexity Institute, Virginia Tech, Blacksburg, VA, United States of America; London School of Hygiene & Tropical Medicine, UNITED KINGDOM

## Abstract

The study objective is to estimate the epidemiological and economic impact of vaccine interventions during influenza pandemics in Chicago, and assist in vaccine intervention priorities. Scenarios of delay in vaccine introduction with limited vaccine efficacy and limited supplies are not unlikely in future influenza pandemics, as in the 2009 H1N1 influenza pandemic. We simulated influenza pandemics in Chicago using agent-based transmission dynamic modeling. Population was distributed among high-risk and non-high risk among 0–19, 20–64 and 65+ years subpopulations. Different attack rate scenarios for catastrophic (30.15%), strong (21.96%), and moderate (11.73%) influenza pandemics were compared against vaccine intervention scenarios, at 40% coverage, 40% efficacy, and unit cost of $28.62. Sensitivity analysis for vaccine compliance, vaccine efficacy and vaccine start date was also conducted. Vaccine prioritization criteria include risk of death, total deaths, net benefits, and return on investment. The risk of death is the highest among the high-risk 65+ years subpopulation in the catastrophic influenza pandemic, and highest among the high-risk 0–19 years subpopulation in the strong and moderate influenza pandemics. The proportion of total deaths and net benefits are the highest among the high-risk 20–64 years subpopulation in the catastrophic, strong and moderate influenza pandemics. The return on investment is the highest in the high-risk 0–19 years subpopulation in the catastrophic, strong and moderate influenza pandemics. Based on risk of death and return on investment, high-risk groups of the three age group subpopulations can be prioritized for vaccination, and the vaccine interventions are cost saving for all age and risk groups. The attack rates among the children are higher than among the adults and seniors in the catastrophic, strong, and moderate influenza pandemic scenarios, due to their larger social contact network and homophilous interactions in school. Based on return on investment and higher attack rates among children, we recommend prioritizing children (0–19 years) and seniors (65+ years) after high-risk groups for influenza vaccination during times of limited vaccine supplies. Based on risk of death, we recommend prioritizing seniors (65+ years) after high-risk groups for influenza vaccination during times of limited vaccine supplies.

## Author summary

The study objective is to estimate the epidemiological and economic impact of vaccine interventions during an influenza pandemic in Chicago, to assist in vaccine intervention priorities. Population dynamics play an important role in influenza pandemic planning and response. To optimally allocate limited vaccine resources, it is important to inform decision makers and public health officials about both the direct benefit among vaccinated population and the indirect benefit among non-vaccinated population. This study adds to the evidence of prior studies by using a detailed agent-based model for estimating the direct and indirect benefits of epidemiological and economic impact of vaccine-based interventions. This study can be extended to analyze for a range of vaccine compliance and efficacy values at different attack rates of influenza pandemics in different rural and urban areas of the United States and at the country level, to infer objective prioritization criteria for influenza vaccine interventions among different risk and age groups.

## Introduction

The Advisory Committee on Immunization Practices (ACIP) recommends seasonal influenza vaccination annually for individuals aged 6 months and older without contraindications to prevent and control seasonal and pandemic influenza [1]. They update information on the dosage for children, antigenic composition and influenza vaccine products. While the ACIP recommendations for 2015–2016 influenza season partially account for risk of transmission, such as influenza immunized individuals caring for immunosuppressed persons are recommended to avoid contact with such persons for 7 days after vaccination, they do not address prioritization of influenza vaccination among subpopulations [2]. For the 2009–2010 influenza pandemic season, the ACIP recommended seasonal influenza vaccination for children above 6 months, adolescents and adults with a focus on individuals at higher risk of influenza complications, or are close contacts of persons at higher risk [3]. In February 2010, ACIP expanded the recommendation of annual influenza vaccination to any person aged above 6 months who does not have contraindications to vaccination, taking effect from the 2010–2011 influenza season. It took months to develop and distribute the 2009 H1N1 influenza vaccine. Similar scenarios of delay in vaccine introduction with limited vaccine efficacy and limited supplies are not unlikely in future influenza pandemics. Understanding and analysis of these challenging scenarios through computational modeling and simulation to improve influenza prevention and control programs is the primary motivation of this study.

### Prioritization of influenza vaccine intervention

Evidence on the epidemiological and economic impact of vaccination for all age and risk groups from the societal standpoint assists in prioritization of influenza vaccine intervention, especially when vaccine supplies are limited, and minimize the direct cost of clinical care for influenza related health outcomes and indirect cost of productivity loss due to workplace absenteeism. While some studies have analyzed the direct epidemiological and economic impact of vaccine intervention strategies on controlling influenza pandemics [4–13], other studies have analyzed both the direct and indirect epidemiological and economic impact of influenza vaccination [14–16]. There are also prior studies that focused on the prioritization of vaccination and other interventions among people in different age groups [17–19]. This study adds to the evidence of prior studies by using a detailed agent-based model for estimating the direct and indirect effects of epidemiological and economic impact of vaccine-based interventions. The objective of the vaccine interventions is to minimize deaths, hospitalizations, outpatient visits, and the number of ill people who do not seek medical care.

### Direct epidemiological and economic effects

Direct effect is due to the immune protection gained by effectively vaccinated individuals, and indirect effect is due to blocking of the influenza transmission by vaccinated individuals to susceptible individuals in their social network. Cost effectiveness of influenza vaccination for 65+ years [5], healthy working adults [6,7], and children [9,20] with a focus on direct effects have been studied. Prosser et al. evaluate the economic impact of 2009 pandemic influenza vaccine intervention for all age and risk groups [8]. They infer that vaccination of the subpopulation with a high risk of developing influenza related complications in each age group is cost saving, and vaccination of the healthy subpopulation in each age group is cost effective. Other studies have inferred that vaccine administration during previous and potential pandemics produces health benefits in terms of number of averted influenza cases and related health outcomes [10–12]. These studies included the direct cost of hospitalizations, outpatient visits, and deaths, and included the related costs of vaccine production and administration, and lost productivity. Depending on the risk and age group of the subpopulations, geographic region, and analytic methodology, the vaccine interventions may or may not be cost effective [21,22].

### Indirect epidemiological and economic effects

Indirect effects account for the indirect protection due to vaccine intervention. Effectively vaccinated individuals who develop protective immune response to the prevalent influenza strains, cut off transmission pathways to secondary and subsequent individuals. The indirect effect of vaccinating school children has been found to be significant, due to their high connectivity in the social network and significance of their transmission pathways to their households and community [23–27]. While Medlock et al. recommend influenza vaccine prioritization of school children and adults aged 30 to 39 years [15], Lee et al. recommend prioritization of vaccinating at-risk individuals first rather than children first by analyzing the 2009 H1N1 influenza pandemic, matching the 2009 ACIP recommendations [14].

**Fig 1:**
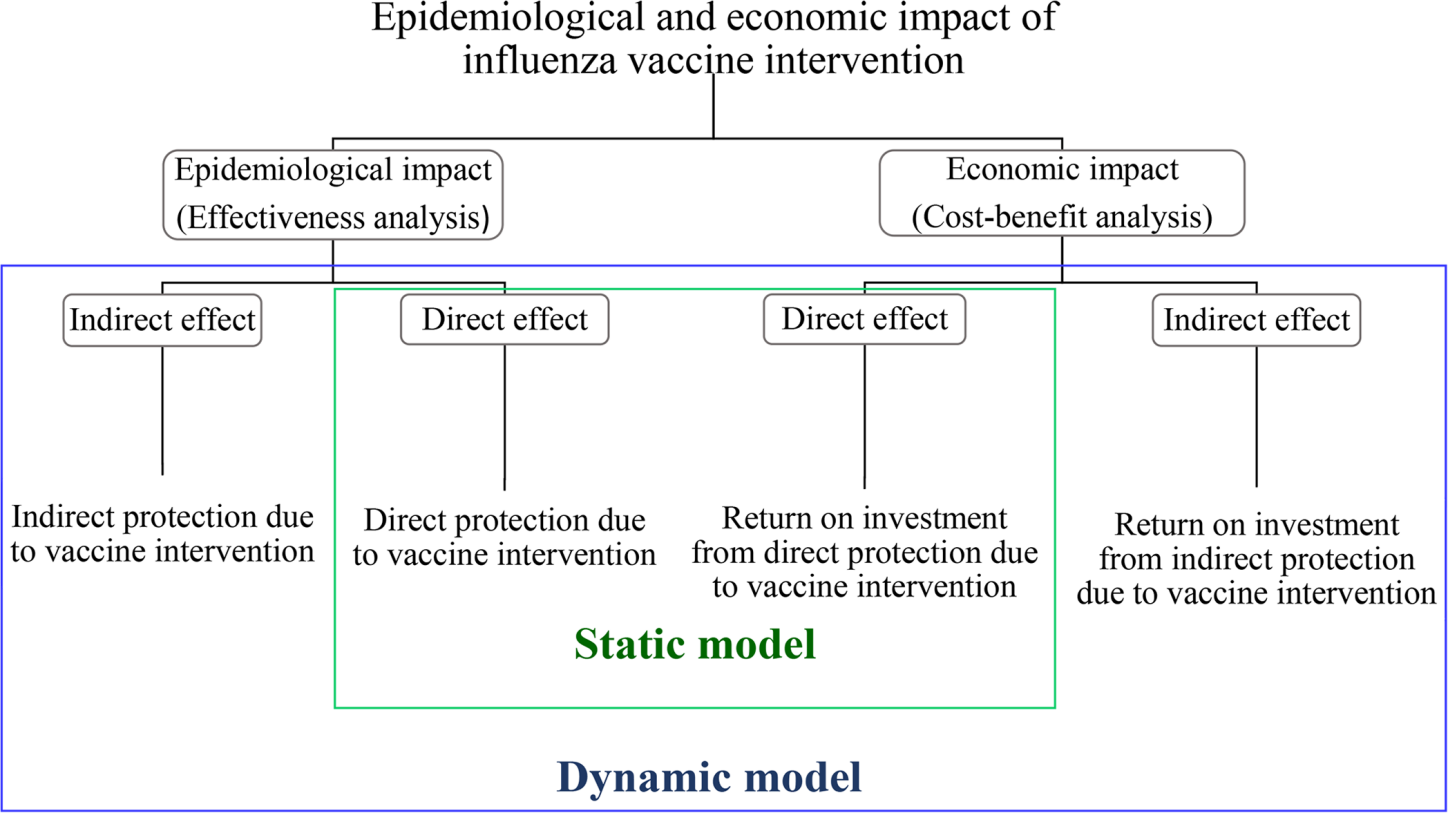
Epidemiological and economic impact of influenza vaccine intervention. The epidemiological and economic impact of influenza vaccine intervention includes the direct and indirect effects. The static model simulated only the direct effects, while the dynamic model simulates both the direct and indirect effects. Direct effect is due to the direct protection of the influenza vaccine among vaccinated individuals who generate protective immune response to influenza infection. Indirect effect is due to indirect protection among non-vaccinated individuals who are protected from influenza acquisition from effectively vaccinated individuals, (i.e.) in the absence of vaccination, influenza transmission will have occurred between these individuals.

The epidemiological benefits and economic costs estimated by taking into account only the direct effect is relatively conservative, in comparison to taking into account both the direct and indirect effects. We improve the fidelity and robustness of the cost-benefit estimates to facilitate optimal prioritization of our vaccine interventions among different age and risk groups. Fig 1 illustrates the evaluation of the epidemiological and economic impact of influenza vaccine intervention using the static model (direct effects only) and dynamic model (direct + indirect effects).

### Study objective

Meltzer et al. estimate the potential net value of different vaccination strategies, and identify vaccination priorities for different age and risk groups during an influenza pandemic [4]. A Monte Carlo based static model is used to estimate the costs and benefits due to the direct effect of vaccine interventions in the United States. We focus our study on similar influenza related health outcomes, risk levels and age groups as Meltzer’s study. We use an agent-based dynamic model to estimate the direct and indirect epidemiological and economic impact of vaccine interventions during an influenza pandemic in Chicago, and assist in the assessment of vaccine intervention priorities.

### Public health significance

Population dynamics play an important role in influenza pandemic planning and response. Influenza vaccination not only protects effectively vaccinated individuals who develop a protective immune response from contracting influenza, but also prevents the spread of influenza in the social contact network of people by breaking the transmission chain. To optimally allocate limited resources, it is important to inform decision makers and public health officials about both the direct and indirect effects of influenza vaccine interventions.

## Methods

### Ethics statement

The Institutional Review Board at Virginia Tech has given ethics approval (IRB exempt) for the research conducted in this study.

### Dynamic agent-based modeling of Chicago synthetic population

The Chicago metropolitan area is a major urban area in the United States, and had high influenza incidence during the 2009 H1N1 influenza pandemic [28]. We analyzed the impact of vaccine-based interventions on pandemic influenza in Chicago, using the population distribution of 9,047,574 people from the census data [29]. The disease diffusion occurs on a collocation based synthetic social contact network for Chicago, based on dynamic agent-based modeling [30–32]. We generated the synthetic population and estimated the social contact network in Chicago through population synthesis, activity assignment, location choice and contact estimation, as illustrated in Table 1 [30,33]. The social contact network simulated the movement of individuals throughout the city and estimated the contact times between individuals based on their simultaneous presence at a location.

**Table 1:** Synthetic social network of Chicago. Synthetic population of Chicago is generated and a social contact network is estimated through the following four steps.

Process	Description
Population synthesis	Synthetic representation of each household in Chicago metropolitan area is created that is statistically identical to US census data when aggregated to a block group level.
Activity assignment	Each synthetic person in a household is assigned a set of activities to perform during the day, along with the times when the activities begin and end, as given by activity or time-use survey data.
Location choice	An appropriate real location is chosen for each activity for every synthetic person based on data such as land use data or Dunn and Bradstreet location data.
Contact estimation	Each synthetic person is deemed to have made contact with a subset of other synthetic people simultaneously present at the same location. This gives rise to the synthetic social contact network

### Influenza transmission dynamics

The transmission dynamics of an influenza-like-illness in the population is simulated using the susceptible-exposed-infectious-recovered (SEIR) epidemiological model on this synthetic social contact network of Chicago. Each person in the model is in one of the following four health states at any time: susceptible, exposed, infectious, and removed. A person is in the susceptible state until he becomes exposed. If a person becomes exposed, he remains exposed for the duration of the latent period, during which he is not infectious. At the end of the latent period, an exposed person becomes infectious and remains infectious for the duration of the infectious period. A person in the infectious state will probabilistically transmit the disease, based on the transmission rate, to any of his contacts who are in the susceptible state. A proportion of infectious individuals are asymptomatic, and there is a reduction in probability of transmission by an asymptomatic infectious person in comparison to a symptomatic infectious person to a susceptible individual. After the infectious period, the infectious person becomes recovered (or removed). Transmissibility is the probability of transmission per minute of contact with a symptomatic infectious person and is set to 0.00008, 0.00009, and 0.0001 to calibrate the simulation for the moderate, strong and catastrophic influenza pandemic scenarios respectively, with attack rates of 11.73%, 21.96% and 30.15% respectively. The simulation parameters for the social contact network and influenza dynamics are illustrated in Table 2. We estimate the direct and indirect effects of vaccine interventions on influenza pandemics of moderate, strong and catastrophic severities, in comparison to the base case scenario of no vaccine intervention.

**Table 2:** Simulation parameters. The parameter values of the influenza pandemic simulations and their sources.

Parameter	Value	Source
Population of Chicago metropolitan area	9,047,574	[28]
Age groups	0–19 yrs, 20–64 yrs, 65+ yrs	[4]
Influenza pandemic severities	Catastrophic, strong, moderate	Simulation calibration
Attack rates of influenza pandemics	30.15% (catastrophic) 21.96% (strong) 11.73% (moderate)	
Transmissibility: Probability of transmission per minute of contact with an symptomatic infectious person	0.0001 (catastrophic) 0.00009 (strong) 0.00008 (moderate)	
Probability of transmission per minute of contact with an asymptomatic infectious person in comparison to a symptomatic infectious person	33%	[50–52]
Proportion of symptomatic infection in influenza infected individuals	67%	Assumed (Proportion of symptomatic infection in influenza infected individuals among healthy participants in studies is 66.9% [53])
Influenza related health outcomes	Death, hospitalization, outpatient visits, ill but not seeking care	[4]
Latent period	1 day [sd: 0.63]	[54,55]
Infectious period	2 days [sd: 1.06]	[54,55]
Serial interval	2.8 days	[56]; Estimated from simulation
Infected individuals at epidemic start (day 0)	100	Assumed
Risk levels among the different age groups	0–19 yrs: 93.6% (non-high), 6.4% (high)	[4]
	20–64 yrs: 85.6% (non-high), 14.4% (high)	
	65+ yrs: 60% (non-high), 40% (high)	
Distribution of influenza related health outcomes among the different age groups and risk levels	See Fig 3	[4]
Cost of influenza vaccine	$28.62	[38]
Medical costs and productivity losses of influenza-related health outcomes among the different age groups	See Table 5	[39]
Efficacy of influenza vaccine	40% Sensitivity analysis: (10%, 20%, 30%, 40%, 50%, 60%)	Assumed (Effectiveness of influenza vaccines varies between 10% to 60% [37])
Vaccine compliance	40% Sensitivity analysis: (10%, 40%, 60%, 80%)	Assumed
Start date of vaccine intervention	15 days after epidemic start Sensitivity analysis: (15, 30, 60, 90)	Assumed
Vaccination period	60 days	Assumed
Vaccine administration rate	60000 people per day	Calibration

### Influenza related health outcomes, risk levels and age groups

Influenza related health outcomes for the infected individuals are *death*, *hospitalization*, *outpatient visits*, and *ill but not seeking medical care*. The risk levels are *high* and *non-high*, and the age groups are *0–19 years*, *20–64 years* and *65+ years*. Based on pre-existing medical conditions, influenza infected individuals may be at a high or non-high risk of experiencing influenza related health outcomes. The distribution of the four influenza related health outcomes among the high and non-high risk cases in the three different age groups is based on the study by Meltzer et al. [4].

### Base case scenario of no vaccine intervention

**Fig 2:**
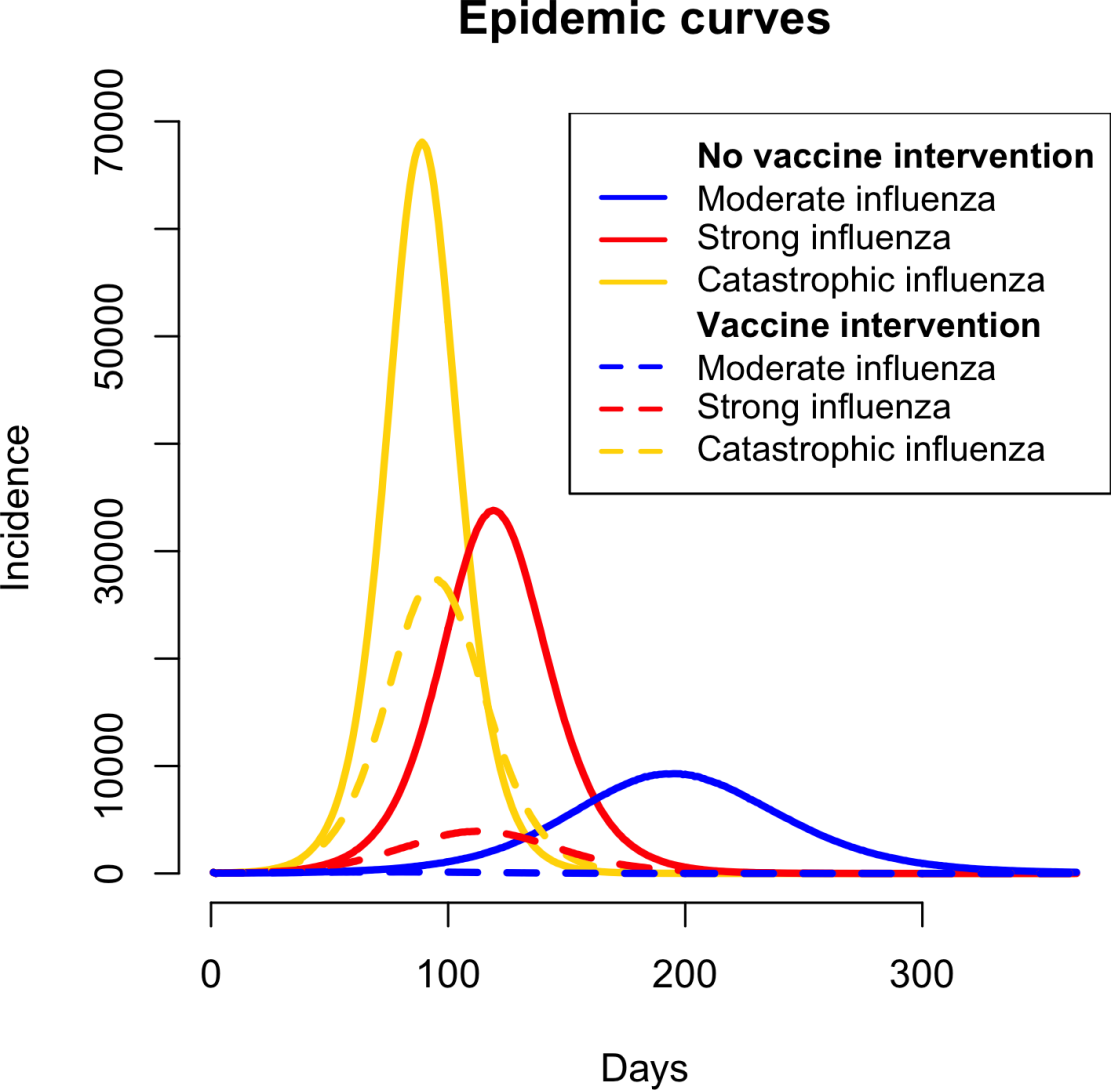
Influenza incidence (average number of new cases per day) during the pandemic for no vaccine intervention and vaccine intervention scenarios. The epidemic curves illustrate influenza incidence without and with vaccination intervention for the catastrophic, strong and moderate influenza pandemic scenarios. The number of cases is the average of new cases over 25 simulations. Higher attack rates cause the earlier, more severe, and shorter pandemic duration, compared to the less severe but longer pandemics. The vaccination intervention is applied 15 days after the start of pandemic and implemented for 60 days. The vaccine intervention scenarios are simulated at 40% efficacy and 40% compliance for all age and risk groups in the dynamic agent-based model.

For the base-case scenario of no vaccine intervention, three different severities of an influenza pandemic were simulated using the dynamic model: moderate influenza with 11.73% attack rate, strong influenza with 21.96% attack rate, and catastrophic influenza with 30.15% attack rate. We use the dynamic model to simulate the epidemic curves for these 3 attack rates for the base-case scenario of no vaccine intervention, based on the average incidence from 25 replicates (see Fig 2 and Table 3). The simulation timeline of the influenza pandemics are in accordance with prior experiences of influenza pandemics in the United States [34]. S1 Appendix describes the risk space of transmissibility and clinical severity for the pandemic scenarios, as defined by the framework for assessing epidemiologic effects of influenza epidemics and pandemics by Reed et al [35,36].

**Table 3:** Pandemic cost per capita, attack rate, and reproduction number for different severities of pandemic influenza in the base case scenario of no vaccine intervention. Pandemic cost per capita is the average cost of influenza related health outcomes among infected individuals for death, hospitalization, outpatient visit, and ill but not seeking medical care. The attack rate is the proportion of population infected by influenza during the influenza pandemic. Reproduction number is the number of secondary cases caused by the index case in a susceptible population.

Base-case scenario of no vaccine intervention	Catastrophic influenza	Strong influenza	Moderate influenza
**Pandemic cost per capita**	$678.10	$486.67	$255.18
**Attack rate**	30.15%	21.96%	11.73%
**Reproduction number**	1.19	1.13	1.06

### Vaccine intervention

Effectiveness of influenza vaccines varies between 10% to 60% [37]. We analyzed the impact of the vaccine intervention scenario of 40% efficacy and 40% compliance for all age and risk groups, following Meltzer et al. [4]. It took months to develop and distribute the 2009 H1N1 influenza vaccine, and similar scenarios of delay in vaccine introduction, limited vaccine efficacy and limited supplies are not unlikely in future influenza pandemics. Thereby, we analyze delays in the implementation of the vaccine intervention with limited efficacy and compliance rates.

### Direct epidemiological effect of vaccine intervention using static model

Static model is used to estimate the direct benefit of influenza vaccination, that is, vaccination only protects effectively vaccinated individuals who develop protective immune response, but does not account for preventing influenza transmission from effectively vaccinated individuals to their social contact network. Using the simulation results of the base-case scenario of no vaccine intervention from the dynamic model, the influenza attack rates of moderate, strong and catastrophic pandemic scenarios are decreased by the proportional impact of the vaccine intervention at 40% coverage and 40% efficacy. Thereby, the influenza attack rates in the 3 age group sub-populations are decreased by 16% (40% efficacy * 40% compliance) in each of the three pandemic scenarios (see Table 4).

**Table 4:** Pandemic cost per capita, attack rate, and reproduction number for catastrophic, strong and moderate pandemic influenza scenarios with and without vaccine intervention. Pandemic cost per capita, attack rate and reproduction number with and without vaccine intervention is presented for catastrophic, strong and moderate influenza pandemic scenarios. The vaccine intervention is implemented at 40% compliance and 40% efficacy which decreases the pandemic cost per capita, attack rate and reproduction number. Pandemic cost per capita, attack rate and reproduction number are relatively lower in the dynamic model (direct + indirect effects) in comparison to the static model (direct effect only).

	No vaccine intervention	Vaccine intervention	
**Catastrophic influenza**	**Base case**	**Static model**	**Dynamic model**
Pandemic cost per capita	$678.10	$581.09	$370.56
Attack rate	30.15% [0–19 years: 48.35%] [20–64 years: 23.94%] [65+ years: 14.91%]	25.33% [0–19 years: 40.62%] [20–64 years: 20.11%] [65+ years: 12.52%]	16.34% [0–19 years: 27.97%] [20–64 years: 12.25%] [65+ years: 7.34%]
Reproduction number	1.19	1.15	1.09
**Strong influenza**	**Base case**	**Static model**	**Dynamic model**
Pandemic cost per capita	$486.67	$420.28	$90.81
Attack rate	21.96% [0–19 years: 36.76%] [20–64 years: 16.82%] [65+ years: 10.18%]	18.45% [0–19 years: 30.88%] [20–64 years: 14.13%] [65+ years: 8.55%]	3.90% [0–19 years: 6.47%] [20–64 years: 2.59%] [65+ years: 1.52%]
Reproduction number	1.13	1.11	1.02
**Moderate influenza**	**Base case**	**Static model**	**Dynamic model**
Pandemic cost per capita	$255.18	$225.83	$14.85
Attack rate	11.73% [0–19 years: 20.64%] [20–64 years:8.55%] [65+ years: 5.05%]	9.85% [0–19 years:17.34%] [20–64 years: 7.19%] [65+ years: 4.24%]	0.16% [0–19 years: 0.30%] [20–64 years: 0.11%] [65+ years: 0.06%]
Reproduction number	1.06	1.05	1.00

### Direct and indirect epidemiological effects of vaccine intervention using dynamic model

We simulated the vaccine intervention scenarios at 40% efficacy and 40% compliance for all age and risk groups in the dynamic agent-based model. The vaccine intervention is initiated 15 days after the start of the pandemic and is carried out for 60 days. The dynamic model simulates the diffusion of influenza on the population in Chicago. It takes into account the indirect effect of limiting disease diffusion by vaccinated individuals, who develop protective immune response and cut off transmission pathways to secondary and subsequent individuals. The influenza attack rates for the 3 age groups in moderate, strong and catastrophic pandemic scenarios are estimated (see Table 4). Fig 2 includes the epidemic curves (based on 25 replicates of each scenario) for the three pandemic scenarios with the vaccine intervention.

### Vaccine cost

The cost of influenza vaccine is estimated to be $28.62, and includes the clinical personnel, non-clinical personnel, and all overhead costs [38]. Direct medical costs and indirect productivity losses were estimated from a prior study, and are presented in Table 5 [39–42].

**Table 5:** Cost of influenza related health outcomes for different age and risk groups. The costs of influenza related health outcomes of death, hospitalization, outpatient, and ill but not seeking medical care are based on the study by Carias et al [39], and are updated to 2015 US dollars.

Influenza related health outcome/ Age group (years)	Medical cost + Productivity losses ($ per person)	
**Death**	**Non-high risk**	**High risk**
0–19	1,640,255	1,650,049
20–64	934,931	941,199
65+	276,971	290,052
**Hospitalization**	**Non-high risk**	**High risk**
0–19	16,883	35,370
20–64	26,345	34,743
65+	14,980	22,478
**Outpatient**	**Non-high risk**	**High risk**
0–19	508	1,051
20–64	634	904
65+	1,282	3,134
**Ill, but not seeking medical care**	**Non-high risk**	**High risk**
0–19	129	129
20–64	88	88
65+	134	134

### Pandemic cost estimation

**Fig 3:**
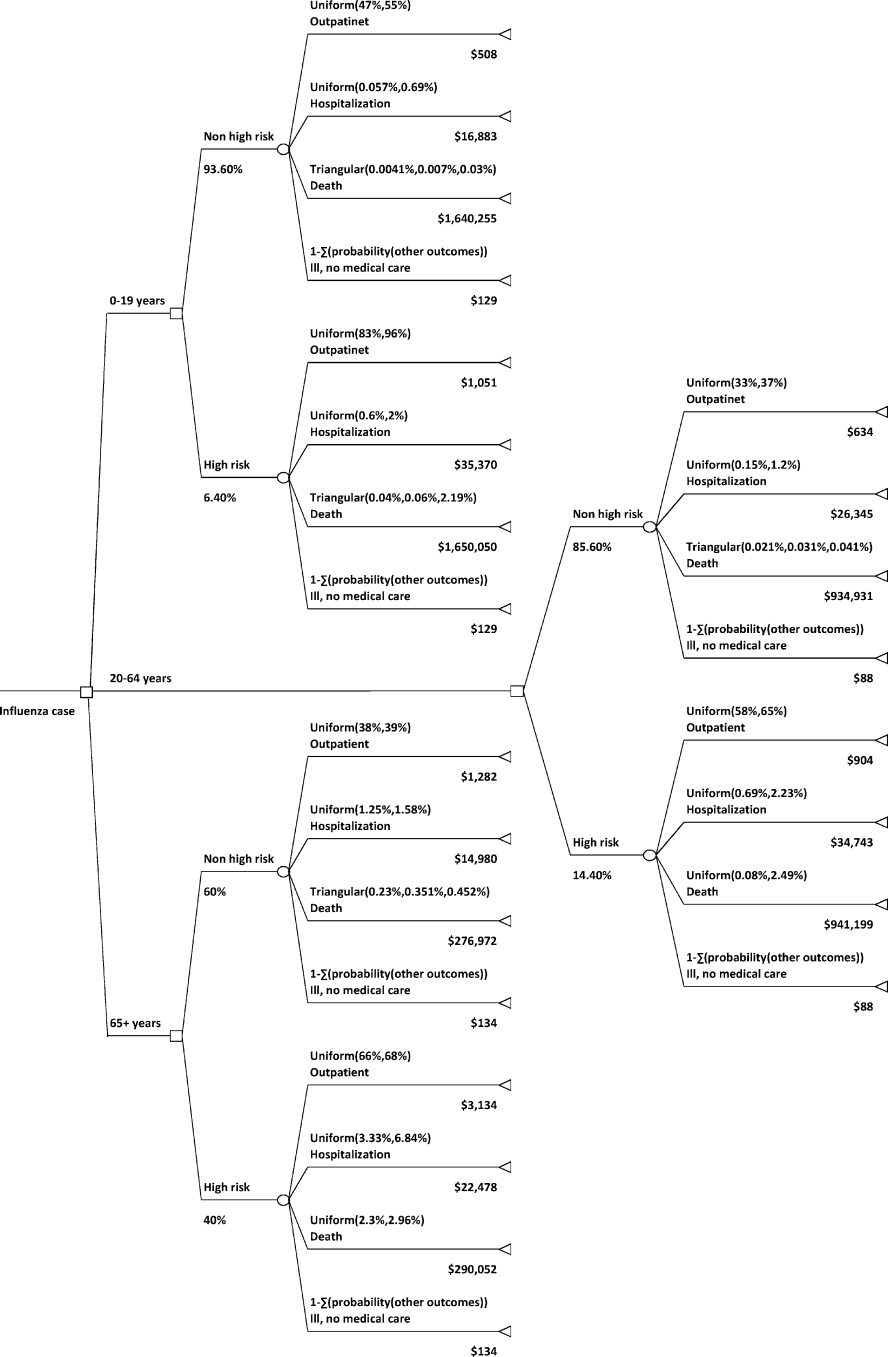
Decision tree of health outcomes for influenza cases and related costs. For each influenza case, the probability of the different health outcomes and related costs depend on the age and risk group of the patient. Patients with pre-existing medical condition have a high risk of experiencing severe influenza related health outcomes. The probability of each health outcome is assigned an uniform or triangular distribution [4]. For the uniform distribution, the lower and upper rate are presented; for triangular distribution, the lower, most probably, and higher rates are presented.

Based on Meltzer’s study [4], we developed a decision tree that includes the probability distribution of an influenza case experiencing the influenza related health outcomes of *death*, *hospitalization*, *outpatient visits*, and *ill but not seeking medical care*, and the cost associated with these health outcomes among the different age and risk groups (see Fig 3). All costs have been adjusted to 2015 US$ (see Table 5). We used this decision tree to estimate the cost due to influenza related health outcomes among the different age and risk groups. This cost estimation process is conducted in all the three scenarios: base case scenario of no intervention using dynamic model, vaccine intervention scenario using static model, and vaccine intervention scenario using dynamic model. Within each of these scenarios, for each pandemic severity (moderate, strong and catastrophic), we compute the *pandemic cost*, *pandemic cost per capita*, *net benefits*, and *return on investment*, as illustrated in Table 6 (also, see Tables 3 and 4). The *pandemic cost* is the total cost associated with the health outcomes of influenza cases and the cost of vaccination, and *pandemic cost per capita* is the average pandemic cost per person. The *net benefits* are the difference in cost due to improved health outcomes from vaccination and the vaccination cost. *Return on investment* is the gain in net benefits relative to the vaccination cost.

**Table 6:** Computation of pandemic cost, pandemic cost per capita, net benefits and return on investment. The formulations to compute *pandemic cost*, *pandemic cost per capita*, *net benefits* and *return on investment* are presented below for the scenarios of without and with vaccine intervention. *Pandemic cost* is the total cost associated with the health outcomes of influenza cases and the cost of vaccination, and *pandemic cost per capita* is the average pandemic cost per person. The *net benefits* is the difference in cost due to improved health outcomes from vaccination and the vaccination cost. *Return on investment* is the gain in net benefits relative to the vaccination cost.

Metrics	No vaccine intervention	Vaccine intervention
	Cost of influenza related health outcomes	(Cost of influenza related health outcomes) + (Vaccination cost)
Pandemic cost	$\left(\sum_{i}\sum_{j}P_{B}^{ij}C_{}^{ij}\right)$	$\left(\sum_{i}\sum_{j}P_{I}^{ij}C_{}^{ij}\right)+(C_{v}N_{v})$
	Per capita (Cost of influenza related health outcomes)	Per capita (Cost of influenza related health outcomes) + Per capita (Vaccination cost)
Pandemic cost per capita	$\frac{\left(\sum_{i}\sum_{j}P_{B}^{ij}C_{}^{ij}\right)}{N}$	$\frac{\left(\sum_{i}\sum_{j}P_{I}^{ij}C_{}^{ij})+(C_{v}N_{v}\right)}{N}$
	*Benefits — Costs* (Benefits from reduction in the cost of influenza related health outcomes due to reduction in influenza cases after vaccine intervention)–(Vaccination cost)	
Net benefits	Not applicable	$\left(\sum_{i}\sum_{j}(P_{B}^{ij}-P_{I}^{ij})C_{}^{ij}\right)-(C_{v}N_{v})$
	$\frac{Net\;benefits}{Vaccination\;cost}$ Return on investment is the gain in net benefits relative to the vaccination cost, that is, dollars saved per $1 investment in vaccine intervention	
Return on investment	Not applicable	$\frac{\left(\sum_{i}\sum_{j}(P_{B}^{ij}-P_{I}^{ij})C_{}^{ij})-(C_{v}N_{v}\right)}{C_{v}N_{v}}$
$P_{B}^{ij}$	Number of infected people of age and risk group *i* with health outcome *j* in the base case scenario of no vaccine intervention	
$P_{I}^{ij}$	Number of infected people of age and risk group *i* with health outcome *j* after vaccine intervention	
***i***	Age and risk groups: 0–19 non-high risk, 0–19 high risk, 20–64 non-high risk, 20–64 high risk, 65+ non-high risk, 65+ high risk	
***j***	Influenza related health outcomes: death, hospitalization, outpatient visit, ill but not seeking medical care	
***C***^***ij***^	Cost of influenza related health outcome *j* for age and risk group *i*	
***C***_***v***_	Influenza vaccine cost	
***N***_***v***_	Number of vaccinated people	
***N***	Total population	

### Perspective of economic evaluation

We conducted economic evaluation from the medical and productivity perspective, and includes the direct cost of clinical care for influenza related health outcomes incurred by the health care provider and indirect cost of productivity loss incurred by the patient. To extend this analysis to a societal perspective, costs incurred by the federal government in vaccine distribution, vaccine coverage monitoring, vaccine effectiveness monitoring, vaccine safety monitoring, health communication, and national coordination and technical assistance [43], productivity loss of volunteers in the influenza vaccine campaign, and costs of global influenza surveillance for vaccine strain selection will need to be included, which are beyond the scope of this study.

### Simulation replicates

The values of the simulation parameters and their sources are shown in Table 2. Each influenza pandemic scenario in the agent-based model is simulated 25 times. The costs of influenza-related health outcomes among the different age and risk groups are estimated using the decision tree (Fig 3). The agent-based model is executed through SIBEL [44], a web-based tool to conduct epidemiological disease studies based on realistic social network simulation, and the influenza-related health outcome estimation using decision tree and cost-benefit analysis is executed through the R software for statistical computing and graphics [45].

### Sensitivity analysis

We conducted univariate sensitivity analysis for vaccine compliance, vaccine efficacy and vaccine start date, and their impact on attack rates and return on investment for catastrophic, strong, and moderate influenza pandemic scenarios with no vaccine intervention (base case), and with vaccine intervention in static model (direct effect) and dynamic model (direct + indirect effects).

## Results

### Base case scenario of no vaccine intervention

The pandemic cost per capita is $678.10, $486.67 and $255.18 for catastrophic, strong, and moderate influenza scenarios respectively (see Table 3). The attack rate is 30.15%, 21.96% and 11.73% for catastrophic, strong, and moderate influenza scenarios respectively. The reproduction number is 1.19, 1.13 and 1.06 for catastrophic, strong, and moderate influenza scenarios respectively. The pandemic cost per capita is positively correlated with attack rate and reproduction number, with the highest in catastrophic influenza scenario followed by the strong and moderate influenza scenarios.

### Vaccine interventions

The vaccine intervention is simulated at 40% compliance and 40% efficacy, using the static model and the dynamic model. The vaccine intervention decreases the pandemic cost per capita, attack rate and reproduction number in the catastrophic, strong and moderate influenza pandemic scenarios in both the static and dynamic models.

**Fig 4:**
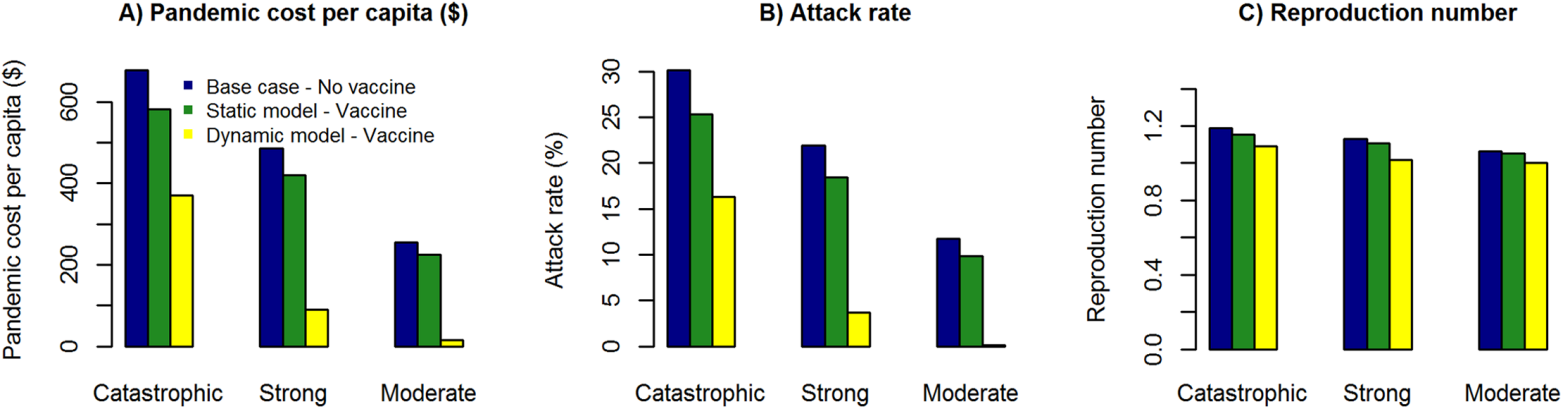
Pandemic cost per capita, attack rate and reproduction number in the catastrophic, strong and moderate influenza pandemic scenarios with and without vaccine intervention. Pandemic cost per capita, attack rate and reproduction number are relatively lower in the dynamic model due to the combined impact of direct and indirect effects, in comparison to the static model which includes only the direct effect. **Fig 4A:** Pandemic cost per capita in the catastrophic, strong and moderate influenza pandemic scenarios with and without vaccine intervention. **Fig 4B:** Attack rate in the catastrophic, strong and moderate influenza pandemic scenarios with and without vaccine intervention. **Fig 4C:** Reproduction number in the catastrophic, strong and moderate influenza pandemic scenarios with and without vaccine intervention.

Fig 4A, 4B and 4C illustrate the comparison of pandemic cost per capita, attack rate and reproduction number in the catastrophic, strong and moderate influenza pandemic scenarios with and without vaccine intervention. In the catastrophic influenza pandemic scenario with vaccine intervention, the pandemic cost per capita, attack rate and reproduction number are $370.56, 16.34% and 1.09 respectively in the dynamic model, while they are $581.09, 25.33% and 1.15 respectively in the static model (see Table 4). In the strong influenza pandemic scenario with vaccine intervention, the pandemic cost per capita, attack rate and reproduction number are $90.81, 3.90% and 1.02 respectively in the dynamic model, while they are $420.28, 18.45% and 1.11 respectively in the static model. In the moderate influenza pandemic scenario with vaccine intervention, the pandemic cost per capita, attack rate and reproduction number are $14.85, 0.16% and 1.00 respectively in the dynamic model, while they are $225.83, 9.85% and 1.05 respectively in the static model.

Molinari et al estimated the annual economic impact (medical costs and productivity loss) of seasonal influenza in the United States to be $87.0673 billion (95% CI: $47.2153, $149.5086) in 2003 with the vaccine intervention [40], which relates to an inflation adjusted cost per capita of $392.24 (95% CI: $212.71, $673.54) in $2015. We estimated the pandemic cost per capita with no vaccine intervention to be $678.10, $486.67 and $255.18 (in $2015) for catastrophic, strong, and moderate influenza scenarios respectively, and with vaccine intervention to be $370.56, $90.81 and $14.85 respectively.

### Direct and indirect effects on return on investment

**Fig 5:**
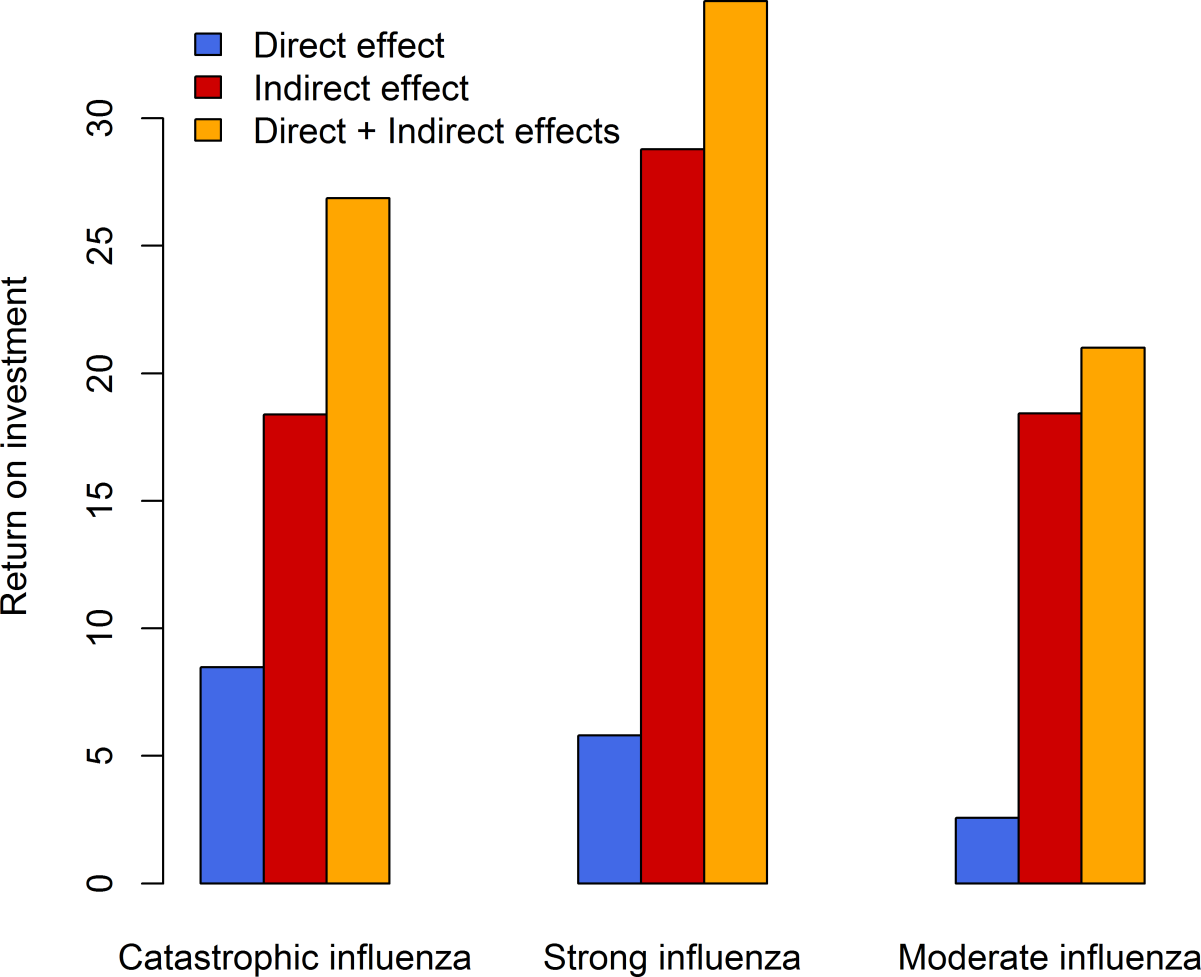
Return on investment of vaccine intervention. Return on investment is the gain in net benefits relative to the vaccination cost, that is, dollars saved per $1 investment in vaccine intervention. Economic impact of the vaccine intervention includes both the direct and indirect effects. The direct effect is evaluated from the static model, and the direct and indirect effects is evaluated from the dynamic model.

While the vaccine interventions are cost-beneficial in both the dynamic and static models, the return on investment is relatively higher in the dynamic model due to the combined impact of direct and indirect effects, in comparison to the static model which includes only the direct effect (see Fig 5 and Table 7).

**Table 7:** Pandemic cost, net benefits and return on investment. *Pandemic cost* is the total cost associated with the health outcomes of influenza cases and the cost of vaccination. N*et benefits* are the difference in cost due to improved health outcomes from vaccination and the vaccination cost. *Return on investment* is the gain in net benefits relative to the vaccination cost, that is, dollars saved per $1 investment in vaccine intervention.

**Static model (direct effect)**					
Pandemic influenza	Pandemic cost with no vaccine intervention (million $)	Pandemic cost with vaccine intervention (million $)	Vaccination cost (million $)	Net benefits (million $)	Return on investment of vaccine intervention
**Catastrophic**	6,135.18	5,153.93	103.56	877.68	8.47
**Strong**	4,403.21	3,698.98	103.57	600.66	5.80
**Moderate**	2,308.79	1,939.60	103.57	265.62	2.56
**Dynamic model (direct + indirect effects)**					
Pandemic influenza	Pandemic cost with no vaccine intervention (million $)	Pandemic cost with vaccine intervention (million $)	Vaccination cost (million $)	Net benefits (million $)	Return on investment of vaccine intervention
**Catastrophic**	6,135.18	3,249.14	103.56	2,782.47	26.87
**Strong**	4,403.21	718.04	103.57	3,581.59	34.58
**Moderate**	2,308.79	30.79	103.57	2174.43	21.00

### Prioritization of vaccine intervention

Vaccine prioritization criteria includes *risk of death*, *total deaths*, *net benefits*, and *return on investment*. Table 8 shows the values for risk of death, total deaths, net benefits, and return on investment of *high* and *non-high risk* groups among the *0–19*, *20–64*, *65+ years* subpopulations for the catastrophic, strong and moderate influenza pandemic scenarios. The prioritization criteria of risk of death, total deaths, net benefits, and return on investment assist in the decision making process for vaccine prioritization among different age and risk groups, as shown in Table 9.

**Table 8:** Risk of death, total deaths, net benefits and return on investment for different age and risk groups in the catastrophic, strong, and moderate influenza pandemic scenarios. *Risk of death* is estimated based on the number of influenza related deaths per 100,000 subpopulation for the specific age and risk groups. *Total deaths* is estimated based on the proportion of influenza related deaths for the specific age and risk groups among total influenza related deaths. *Net benefits* are the difference in cost due to improved health outcomes from vaccination and the vaccination cost. *Return on investment* is the gain in net benefits relative to the vaccination cost, that is, dollars saved per $1 investment in vaccine intervention.

**Catastrophic influenza pandemic**				
**Age and risk group**	**Risk of death** **(per 100,000 cases)**	**Proportion of total deaths**	**Net benefits (million $)**	**Return on investment**
Non-high risk 0–19 yrs	6.63	0.031	280.05	9.86
High risk 0–19 yrs	369.70	0.117	484.16	249.16
Non-high risk 20–64 yrs	7.43	0.065	436.93	8.20
High risk 20–64 yrs	308.19	0.452	1201.38	133.99
Non-high risk 65+ yrs	51.36	0.055	68.80	10.49
High risk 65+ yrs	392.18	0.280	311.13	47.41
**Strong influenza pandemic**				
**Age and risk group**	**Risk of death** **(per 100,000 cases)**	**Proportion of total deaths**	**Net benefits (million $)**	**Return on investment**
Non-high risk 0–19 yrs	5.04	0.033	425.90	14.99
High risk 0–19 yrs	281.08	0.127	714.00	367.42
Non-high risk 20–64 yrs	5.22	0.064	542.70	10.18
High risk 20–64 yrs	216.44	0.450	1462.52	163.11
Non-high risk 65+ yrs	35.08	0.053	79.71	12.15
High risk 65+ yrs	267.86	0.272	356.77	54.37
**Moderate influenza pandemic**				
**Age and risk group**	**Risk of death** **(per 100,000 cases)**	**Proportion of total deaths**	**Net benefits (million $)**	**Return on investment**
Non-high risk 0–19 yrs	2.83	0.036	279.50	9.83
High risk 0–19 yrs	157.85	0.139	483.30	248.69
Non-high risk 20–64 yrs	2.65	0.064	300.61	5.64
High risk 20–64 yrs	110.11	0.447	864.83	96.45
Non-high risk 65+ yrs	17.38	0.052	43.02	6.56
High risk 65+ yrs	132.71	0.263	203.17	30.96

**Table 9:** Prioritization of influenza vaccine intervention. Prioritization of influenza vaccine intervention among different age and risk groups based on different criteria: *risk of death*, *total deaths*, *net benefits*, and *return on investment*. ^**a**^*Risk of death* is estimated based on the number of influenza related deaths per 100,000 subpopulation for the specific age and risk groups. Risk of death is the highest among the high risk 65+ years subpopulation in the catastrophic influenza and it is the highest among high risk 0–19 years subpopulation in the strong, and moderate influenza pandemic scenarios. ^**b**^*Total deaths* is estimated based on the proportion of influenza related deaths for the specific age and risk groups among total influenza related deaths. The proportion of influenza related deaths is the highest among the high risk 20–64 years subpopulation in the catastrophic, strong, and moderate influenza pandemic scenarios. ^**c**^*Net benefits* are the difference in cost due to improved health outcomes from vaccination and the vaccination cost. Net benefits are the highest among the high risk 20–64 years subpopulation in the catastrophic, strong, and moderate influenza pandemic scenarios. ^**d**^*Return on investment* is the gain in net benefits relative to the vaccination cost, that is, dollars saved per $1 investment in vaccine intervention. Return on investment is highest among the high risk 0–19 years subpopulation in the catastrophic, strong and moderate influenza pandemic scenarios.

**Prioritization criteria–Catastrophic influenza pandemic**				
**Priority**	**Risk of death**^**a**^	**Total deaths**^**b**^	**Net benefits**^**c**^	**Return on investment**^**d**^
**1** (high)	High risk 65+ yrs	High risk 20–64 yrs	High risk 20–64 yrs	High risk 0–19 yrs
**2**	High risk 0–19 yrs	High risk 65+ yrs	High risk 0–19 yrs	High risk 20–64 yrs
**3**	High risk 20–64 yrs	High risk 0–19 yrs	Non-high risk 20–64 yrs	High risk 65+ yrs
**4**	Non-high risk 65+ yrs	Non-high risk 20–64 yrs	High risk 65+ yrs	Non-high risk 65+ yrs
**5**	Non-high risk 20–64 yrs	Non-high risk 65+ yrs	Non-high risk 0–19 yrs	Non-high risk 0–19 yrs
**6** (low)	Non-high risk 0–19 yrs	Non-high risk 0–19 yrs	Non-high risk 65+ yrs	Non-high risk 20–64 yrs
**Prioritization criteria–Strong influenza pandemic**				
**Priority**	**Risk of death**	**Total deaths**	**Net benefits**	**Return on investment**
**1** (high)	High risk 0–19 yrs	High risk 20–64 yrs	High risk 20–64 yrs	High risk 0–19 yrs
**2**	High risk 65+ yrs	High risk 65+ yrs	High risk 0–19 yrs	High risk 20–64 yrs
**3**	High risk 20–64 yrs	High risk 0–19 yrs	Non-high risk 20–64 yrs	High risk 65+ yrs
**4**	Non-high risk 65+ yrs	Non-high risk 20–64 yrs	Non-high risk 0–19 yrs	Non-high risk 0–19 yrs
**5**	Non-high risk 20–64 yrs	Non-high risk 65+ yrs	High risk 65+ yrs	Non-high risk 65+ yrs
**6** (low)	Non-high risk 0–19 yrs	Non-high risk 0–19 yrs	Non-high risk 65+ yrs	Non-high risk 20–64 yrs
**Prioritization criteria–Moderate influenza pandemic**				
**Priority**	**Risk of death**	**Total deaths**	**Net benefits**	**Return on investment**
**1** (high)	High risk 0–19 yrs	High risk 20–64 yrs	High risk 20–64 yrs	High risk 0–19 yrs
**2**	High risk 65+ yrs	High risk 65+ yrs	High risk 0–19 yrs	High risk 20–64 yrs
**3**	High risk 20–64 yrs	High risk 0–19 yrs	Non-high risk 20–64 yrs	High risk 65+ yrs
**4**	Non-high risk 65+ yrs	Non-high risk 20–64 yrs	Non-high risk 0–19 yrs	Non-high risk 0–19 yrs
**5**	Non-high risk 0–19 yrs	Non-high risk 65+ yrs	High risk 65+ yrs	Non-high risk 65+ yrs
**6** (low)	Non-high risk 20–64 yrs	Non-high risk 0–19 yrs	Non-high risk 65+ yrs	Non-high risk 20–64 yrs

### Risk of death

**Fig 6:**
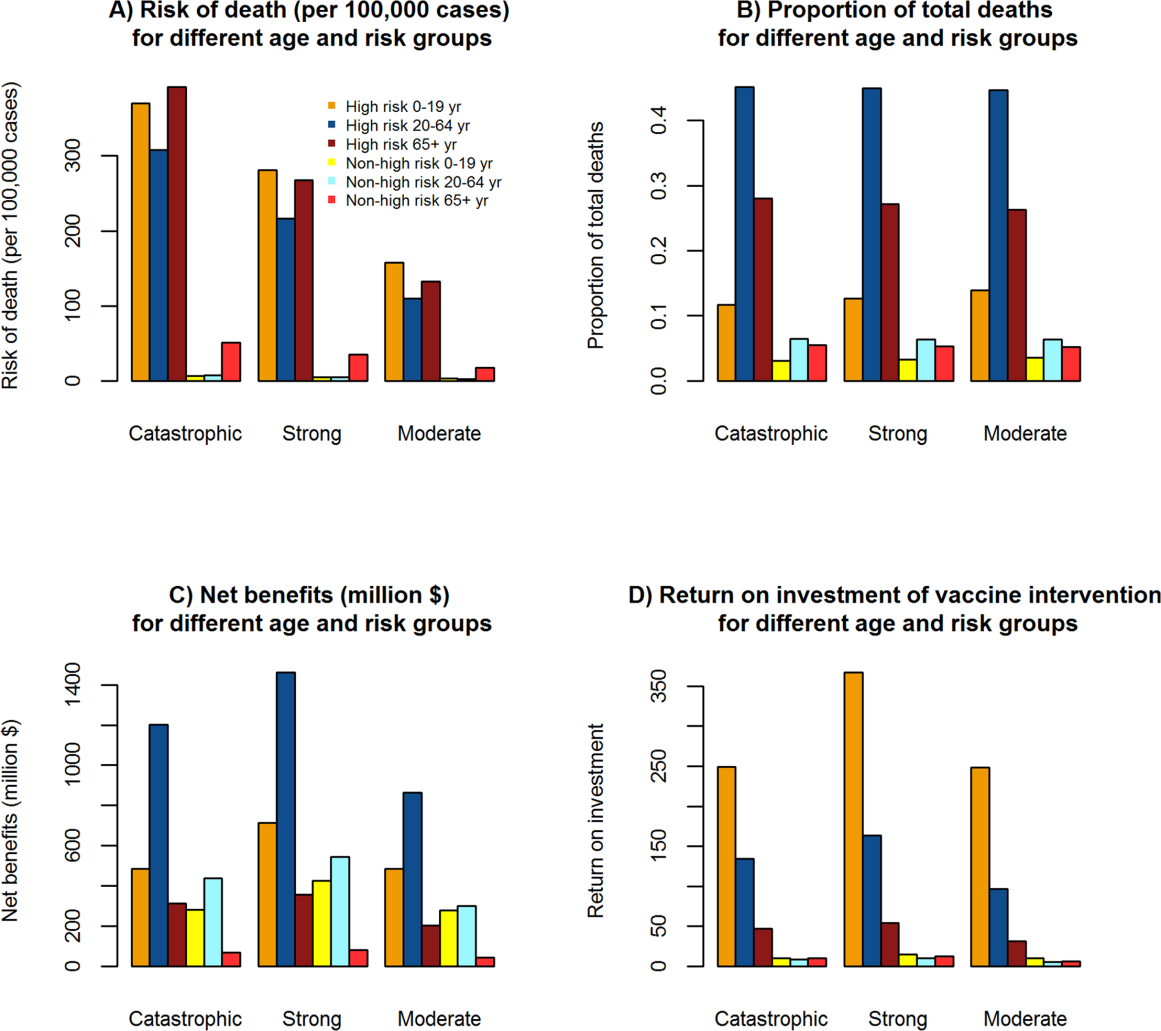
Prioritization of influenza vaccine intervention. Prioritization of influenza vaccine intervention among different age and risk groups based on different criteria: *risk of death*, *total deaths*, *net benefits*, and *return on investment*. **Fig 6A:** Risk of death is estimated based on the number of influenza related deaths per 100,000 subpopulation for the specific age and risk groups. Risk of death is the highest among the high risk 65+ years subpopulation in the catastrophic influenza and it is the highest among high risk 0–19 years old among strong, and moderate influenza pandemic scenarios. **Fig 6B:** Total deaths is estimated based on the proportion of influenza related deaths for the specific age and risk groups among total influenza related deaths. The proportion of influenza related deaths is the highest among the high risk 20–64 years subpopulation in the catastrophic, strong, and moderate influenza pandemic scenarios. **Fig 6C:** Net benefits are the difference in cost due to improved health outcomes from vaccination and the vaccination cost. Net benefits are the highest among the high risk 20–64 years subpopulation in the catastrophic, strong, and moderate influenza pandemic scenarios. **Fig 6D:** Return on investment is the gain in net benefits relative to the vaccination cost, that is, dollars saved per $1 investment in vaccine intervention. Return on investment is highest among the high risk 0–19 years subpopulation in the catastrophic, strong and moderate influenza pandemic scenarios.

Fig 6A illustrates the prioritization criteria for the vaccine intervention based on the *risk of death*. In the catastrophic influenza pandemic scenario, the risk of death among the high-risk 65+ years subpopulation is the highest at 392.18 deaths per 100,000 influenza cases, while it is the lowest among the non-high risk 0–19 years subpopulation at 6.63 deaths per 100,000 influenza cases. In the strong influenza pandemic scenario, the risk of death among the high-risk 0–19 years subpopulation is the highest at 281.08 deaths per 100,000 influenza cases, while it is the lowest among the non-high risk 0–19 years subpopulation at 5.04 deaths per 100,000 influenza cases. In the moderate influenza pandemic scenario, the risk of death among the high-risk 0–19 years subpopulation is the highest at 157.85 deaths per 100,000 influenza cases, while it is the lowest among the non-high risk 20–64 years subpopulation at 2.65 deaths per 100,000 influenza cases.

### Total deaths

Fig 6B illustrates the prioritization criteria for the vaccine intervention based on the proportion of *total deaths*. In the catastrophic influenza pandemic scenario, the proportion of total deaths among the high-risk 20–64 years subpopulation is the highest at 0.45, while it is the lowest among the non-high risk 0–19 years subpopulation at 0.031. In the strong influenza pandemic scenario, the proportion of total deaths among the high-risk 20–64 years subpopulation is the highest at 0.45 while it is the lowest among the non-high risk 0–19 years subpopulation at 0.033. In the moderate influenza pandemic scenario, the proportion of total deaths among the high risk 20–64 years subpopulation is the highest at 0.447, while it is the lowest among the non-high risk 0–19 years subpopulation at 0.036.

### Net benefits

Fig 6C illustrates the prioritization criteria for the vaccine intervention based on *net benefits*. In the catastrophic influenza pandemic scenario, the net benefits among the high-risk 20–64 years subpopulation is the highest at $1201.38 million, while it is the lowest among the non-high risk 65+ years subpopulation at $68.80 million. In the strong influenza pandemic scenario, the net benefits among the high risk 20–64 years subpopulation is the highest at $1462.52 million, while it is the lowest among the non-high risk 65+ years subpopulation at $79.71 million. In the moderate influenza pandemic scenario, the net benefits among the high risk 20–64 years subpopulation is the highest at $864.83 million, while it is the lowest among the non-high risk 65+ years subpopulation at $43.02 million.

### Return on investment

Fig 6D illustrates the prioritization criteria for the vaccine intervention based on *return on investment*. In the catastrophic influenza pandemic scenario, the return on investment among the high-risk 0–19 years subpopulation is the highest at 249.16 (i.e., $249.16 saved for every $1 invested in vaccine intervention), while it is the lowest among the non-high risk 20–64 years subpopulation at 8.20 (i.e., $8.20 saved for every $1 invested in vaccine intervention). In the strong influenza pandemic scenario, the return on investment among the high-risk 0–19 years subpopulation is the highest at 367.42 (i.e., $367.42 saved for every $1 invested in vaccine intervention), while it is the lowest among the non-high risk 20–64 years subpopulation at 10.18 (i.e., $10.18 saved for every $1 invested in vaccine intervention). In the moderate influenza pandemic scenario, the return on investment among the high-risk 0–19 years subpopulation is the highest at 248.69 (i.e., $248.69 saved for every $1 invested in vaccine intervention), while it is the lowest among the non-high risk 20–64 years subpopulation at 5.64 (i.e., $5.64 saved for every $1 invested in vaccine intervention).

### Sensitivity analysis

We conducted univariate sensitivity analysis for vaccine compliance, vaccine efficacy and vaccine start date, and their impact on attack rates and return on investment for catastrophic, strong, and moderate influenza pandemic scenarios.

#### Vaccine compliance, vaccine efficacy and vaccine start date impact on attack rate

**Fig 7:**
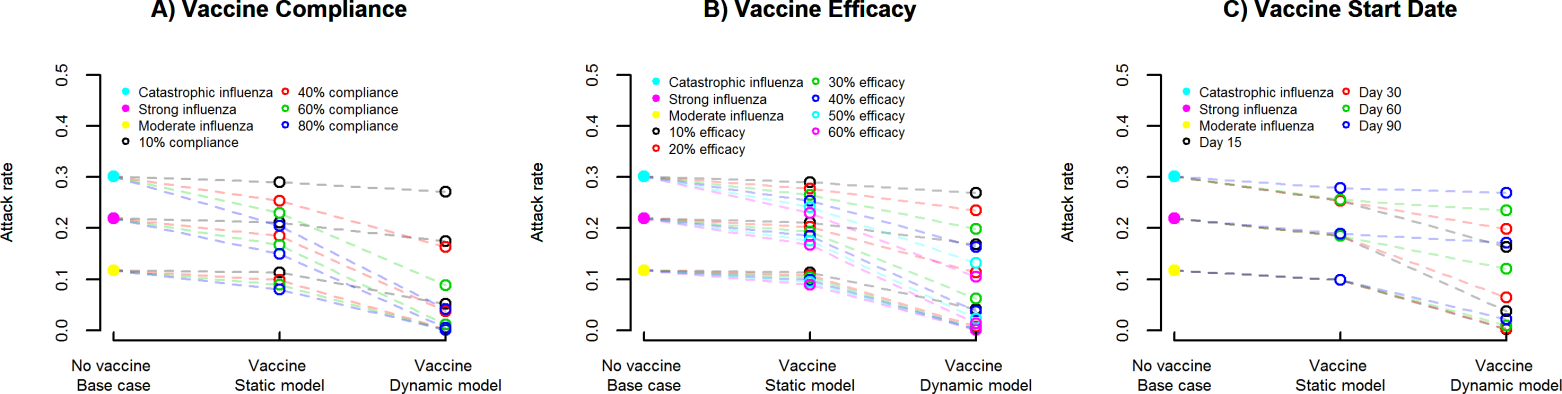
Sensitivity analysis of vaccine compliance, vaccine efficacy and vaccine start date, and impact on attack rate. Univariate sensitivity analysis for vaccine compliance rates of 10%, 40%, 60% and 80% (Fig 7A), vaccine efficacy rates of 10%, 20%, 30%, 40%, 50% and 60% (Fig 7B) and vaccine start dates after epidemic onset of day 15, day 30, day 60 and day 90 (Fig 7C), and their impact on attack rates for catastrophic, strong, and moderate influenza pandemic scenarios with no vaccine intervention (base case) and vaccine intervention (static and dynamic models).

Fig 7 illustrates the univariate sensitivity analysis for vaccine compliance rates of 10%, 40%, 60% and 80% (Fig 7A), vaccine efficacy rates of 10%, 20%, 30%, 40%, 50% and 60% (Fig 7B) and vaccine start dates after epidemic onset of day 15, day 30, day 60 and day 90 (Fig 7C), and their impact on attack rates for catastrophic, strong, and moderate influenza pandemic scenarios with no vaccine intervention (base case) and vaccine intervention (static and dynamic models).

We observe a negative correlation between vaccine compliance and attack rate, negative correlation between vaccine efficacy and attack rate, and positive correlation between vaccine start date and attack rate. The relative impact in the dynamic model is higher due to the combined benefits of direct and indirect effects of the vaccine intervention, in comparison to the static model with only the direct effect of the vaccine intervention.

#### Vaccine compliance impact on return on investment

**Fig 8:**
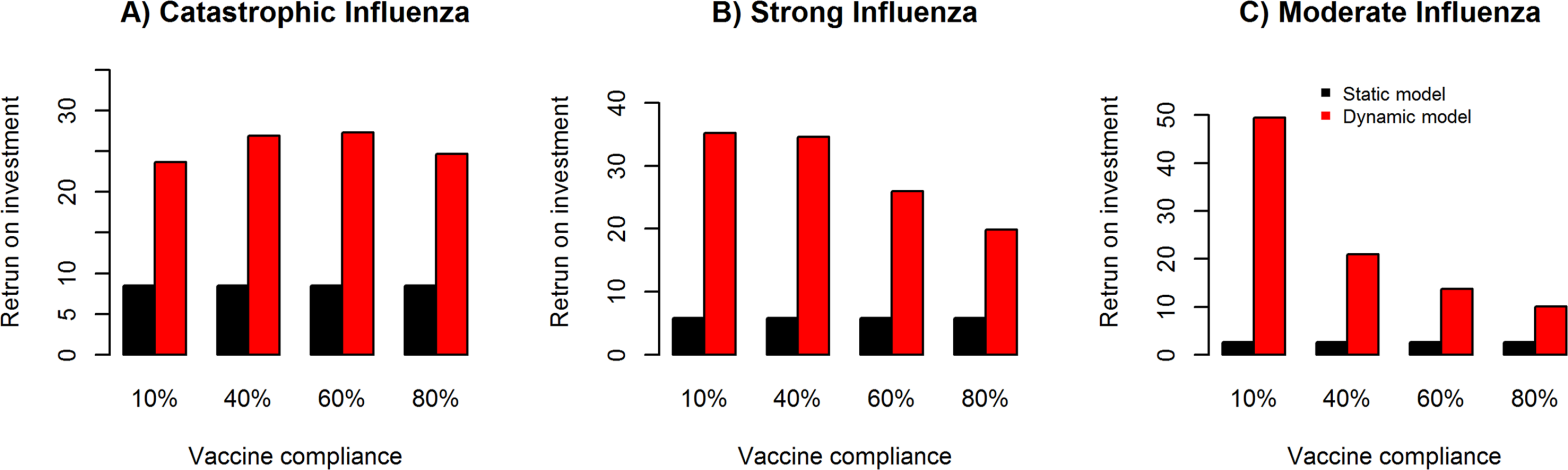
Sensitivity analysis of vaccine compliance and impact on return on investment. Univariate sensitivity analysis for vaccine compliance rates of 10%, 40%, 60% and 80%, and their impact on return on investment for catastrophic (**Fig 8A**), strong (**Fig 8B**) and moderate (**Fig 8C)** influenza pandemic scenario in the static (direct benefit) and dynamic (direct + indirect benefits) models.

Fig 8 illustrates the univariate sensitivity analysis for vaccine compliance rates of 10%, 40%, 60% and 80%, and their impact on return on investment for catastrophic (Fig 8A), strong (Fig 8B) and moderate (Fig 8C) influenza pandemic scenario in the static (direct benefit) and dynamic (direct + indirect benefits) models. We observe a negative correlation between vaccine compliance and return on investment in the dynamic model, indicating that the return on investment is higher with vaccine introduction and decreases with vaccine compliance but also beneficially decreases the attack rate. In the static model, return on investment remains stable for the varied rates of vaccine compliance, with relatively higher return on investment in the catastrophic followed by strong and moderate pandemic influenza scenarios.

#### Vaccine efficacy impact on return on investment

**Fig 9:**
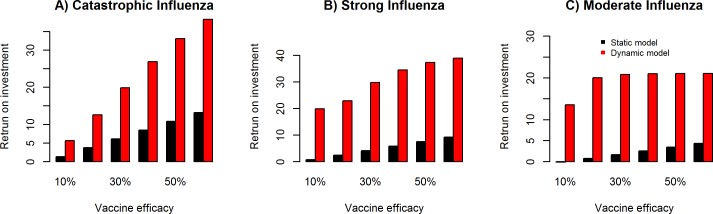
Sensitivity analysis of vaccine efficacy and impact on return on investment. Univariate sensitivity analysis for vaccine efficacy rates of 10%, 20%, 30%, 40%, 50% and 60%, and their impact on return on investment for catastrophic (**Fig 9A**), strong (**Fig 9B**) and moderate (**Fig 9C**) influenza pandemic scenario in the static (direct benefit) and dynamic (direct + indirect benefits) models.

Fig 9 illustrates the univariate sensitivity analysis for vaccine efficacy rates of 10%, 20%, 30%, 40%, 50% and 60%, and their impact on return on investment for catastrophic (Fig 9A), strong (Fig 9B) and moderate (Fig 9C) influenza pandemic scenario in the static and dynamic models. We observe a positive correlation between vaccine efficacy and return on investment in the dynamic and static models, with relatively higher return on investment in the dynamic model compared to the static model.

#### Vaccine start date impact on return on investment

**Fig 10:**
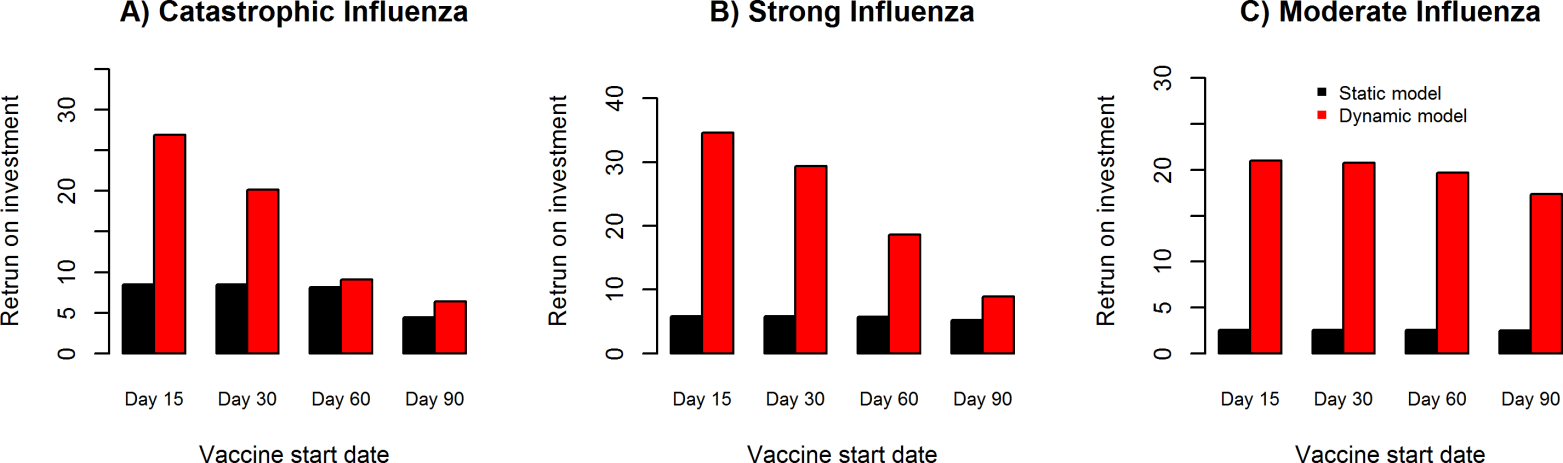
Sensitivity analysis of vaccine start date and impact on return on investment. Univariate sensitivity analysis for vaccine start dates after epidemic onset of day 15, day 30, day 60 and day 90, and their impact on return on investment for catastrophic (**Fig 10A**), strong (**Fig 10B**) and moderate (**Fig 10C**) influenza pandemic scenario in the static (direct benefit) and dynamic (direct + indirect benefits) models.

Fig 10 illustrates the univariate sensitivity analysis for vaccine start dates after epidemic onset of day 15, day 30, day 60 and day 90, and their impact on return on investment for catastrophic (Fig 10A), strong (Fig 10B) and moderate (Fig 10C) influenza pandemic scenario in the static and dynamic models. We observe a negative correlation between vaccine start date and return on investment in the dynamic and static models, with relatively higher return on investment in the dynamic model compared to the static model.

## Discussion

### Direct and indirect epidemiological and economic effects of vaccine intervention

Direct effect is due to the immune protection gained by effectively vaccinated individuals, and indirect effect is due to blocking of the influenza transmission by vaccinated individuals to susceptible individuals in their social network. The static model provides a conservative estimate of the epidemiological and economic benefits of influenza vaccine intervention by accounting for only the direct effect. The dynamic model provides a comprehensive estimate of the epidemiological and economic benefits of influenza vaccine intervention by accounting for both the direct and indirect effects.

The vaccine intervention has a higher probability of effectively vaccinating individuals who will have otherwise being infected in the absence of the vaccine intervention in more severe pandemic scenarios (such as catastrophic influenza). This is due to relatively higher attack rates and higher proportion of population at risk of influenza infection in comparison to less severe pandemic scenarios (such as moderate influenza). Thereby, the impact of the direct effect decreases from catastrophic, strong to moderate influenza pandemic scenarios (see Fig 5).

The vaccine intervention has a lower probability of breaking transmission pathways in more severe pandemic scenarios (such as catastrophic influenza), because the transmission network is densely connected in comparison to sparsely connected transmission networks in less severe pandemic scenarios (such as moderate influenza). Thereby, the impact of the indirect effect increases from catastrophic, strong to moderate influenza pandemic scenarios (see Fig 5).

Pandemic cost per capita, attack rate and reproduction number are relatively lower in the dynamic model due to the combined impact of direct and indirect effects, in comparison to the static model which includes only the direct effect, in the catastrophic, strong and moderate influenza pandemic scenarios. While the vaccine interventions are cost-beneficial in both the dynamic and static models, the return on investment is relatively higher in the dynamic model in comparison to the static model.

### Prioritization of vaccine interventions

We analyzed vaccine prioritization criteria based on *risk of death*, *total deaths*, *net benefits* and *return on investment* for the high and non-high risk groups among 0–19, 20–64 and 65+ years subpopulations. The *risk of death* is the highest among the *high-risk 65+ years* subpopulation in the catastrophic influenza, and it is the highest among the *high-risk 0–19 years* subpopulation in the strong and moderate influenza pandemic scenarios. The proportion of *total deaths* is the highest among the *high-risk 20–64 years* subpopulation in the catastrophic, strong and moderate influenza pandemic scenarios. The *net benefits* are the highest among the *high-risk 20–64 years* subpopulation in the catastrophic, strong and moderate influenza pandemic scenarios. The *return on investment* is the highest in the *high-risk 0–19 years* subpopulation in the catastrophic, strong and moderate influenza pandemic scenarios.

The proportion of total deaths and net benefits measure the epidemiological and economic impact respectively, and are dependent on the absolute size of the different risk and age group subpopulations. Risk of death and return on investment measure the epidemiological and economic impact respectively, and are independent of the absolute size of the different risk and age group subpopulations. Based on risk of death and return on investment, high-risk groups of the three age group subpopulations are recommended for prioritization of influenza vaccine intervention. Also, the vaccine intervention is cost-beneficial for all age and risk groups.

### Targeted vaccination

The attack rates among the children (0–19 years) are higher than the attack rates among the adults (20–64 years) and seniors (65+ years) in the catastrophic, strong, and moderate influenza pandemic scenarios, as illustrated in Table 4. This can be attributed to their larger social contact network and homophilous interactions in schools. Thereby, if we target children for vaccination, there will be higher reduction among the children as well on the overall attack rate in the general population, as also illustrated in prior studies by Hodgson et al [16], Ferguson et al [17], and Germann et al [18]. Also, as shown in Table 9, high risk children have the highest return on investment from the vaccine intervention.

### Public health implications

The dynamic model provides improved estimates of the epidemiological and economic benefits of vaccine interventions in comparison to a static model, by accounting for both the direct and indirect effects. These comprehensive estimates assist in prioritization of vaccine interventions among subpopulations of different risk and age groups, especially during influenza pandemics with limited availability of vaccines. Decision makers can use the dynamic model simulations to compare the epidemiological and economic impact of using different prioritization criteria of influenza vaccine interventions among different risk and age group subpopulations, thereby optimizing allocation of limited resources and improving evidence-based public health policy and practice.

Based on risk of death and return on investment, high-risk groups of the three age group subpopulations can be prioritized for vaccination, and the vaccine interventions are cost saving for all age and risk groups. The attack rates among the children are higher than among the adults and seniors in the catastrophic, strong, and moderate influenza pandemic scenarios, due to their larger social contact network and homophilous interactions in school. Based on return on investment and higher attack rates among children, we recommend prioritizing children (0–19 years) and seniors (65+ years) after high-risk groups for influenza vaccination during times of limited vaccine supplies. Based on risk of death, we recommend prioritizing seniors (65+ years) after high-risk groups for influenza vaccination during times of limited vaccine supplies.

### Modeling implications

We used an agent-based individual model in this study to estimate the direct and indirect epidemiological and economic impact of vaccine interventions during an influenza pandemic in Chicago, similar to related studies [14,46,47]. Alternatively, a population level compartmental model can also be used to conduct this study, similar to related studies [15,48,49]. While agent-based individual models add heterogeneity in contact patterns between individuals in comparison to homogeneous mixing in compartmental models, it will be valuable to compare the vaccine intervention priorities derived from these two modeling methods in future studies.

### Limitations

We used a mean estimate of $28.62 (inflation adjusted to 2015 US dollars) for the cost of influenza vaccine, and did not include the range and uncertainty in vaccination costs by location and size of clinical practice. While beyond the scope of this study, this analysis can be extended to additional studies for a range of vaccine compliance and efficacy values at different attack rates of influenza pandemics in different rural and urban areas of the United States and at the country level, to infer objective prioritization criteria for influenza vaccine interventions among different risk and age groups.

## Supporting information

S1 Appendix Risk space of transmissibility and clinical severity of influenza pandemic. (DOCX)
