# Supplementary material for: Epidemiological and economic impact of pandemic influenza in Chicago: Priorities for vaccine interventions
Source: PLoS Comput Biol. 2017 Jun 1;13(6):e1005521. doi: 10.1371/journal.pcbi.1005521 (PMC5453424; doi:10.1371/journal.pcbi.1005521)
Supplement: S1 Appendix — (DOCX) [file pcbi.1005521.s001.docx]

**S1 Appendix. Risk space of transmissibility and clinical severity of influenza pandemic.**

The risk space of transmissibility and clinical severity for the moderate, strong and catastrophic pandemic scenarios, as defined by the framework for assessing epidemiologic effects of influenza epidemics and pandemics by Reed et al [[35]](https://paperpile.com/c/k8iVzH/WA8A), are illustrated in the table below. Transmissibility is measured on a scale of 1 to 5, and clinical severity is measured on a scale of 1 to 7, with higher values referring to higher transmissibility and clinical severity. The scaled measure of transmissibility is based on symptomatic attack rate in community, symptomatic attack rate in school, symptomatic attack rate in workplace, household secondary attack rate (symptomatic), basic reproductive number (R_0_), and peak outpatient visits for influenza-like illness. The scaled measure of clinical severity is based on case-fatality ratio, case-hospitalization ratio, and ratio of deaths to hospitalization.

| **Pandemic scenario** | **Transmissibility** |
| --- | --- |
| Moderate influenza | 2 |
| Strong influenza | 4 |
| Catastrophic influenza | 5 |
|  | |
| **Age group & risk levels** | **Clinical severity** |
| 0-19 years; non-high risk | 1 |
| 0-19 years; high risk | 2-3 |
| 20-64 years; non-high risk | 2 |
| 20-64 years; high risk | 7 |
| 65+ years; non-high risk | 5 |
| 65+ years; high risk | 7 |
